# Melatonin Inhibits the Progression of Hepatocellular Carcinoma through MicroRNA Let7i-3p Mediated RAF1 Reduction

**DOI:** 10.3390/ijms19092687

**Published:** 2018-09-10

**Authors:** Tong-Hong Wang, Chuen Hsueh, Chin-Chuan Chen, Wan-Syuan Li, Chau-Ting Yeh, Jang-Hau Lian, Junn-Liang Chang, Chi-Yuan Chen

**Affiliations:** 1 Tissue Bank, Chang Gung Memorial Hospital, Tao-Yuan 33305, Taiwan; cellww@gmail.com (T.-H.W); ch9211@cgmh.org.tw (C.H.); chinchuan@mail.cgu.edu.tw (C.-C.C.); apisoulyoyo@gmail.com (W.-S.L.); 2 Graduate Institute of Health Industry Technology and Research Center for Industry of Human Ecology, College of Human Ecology, Chang Gung University of Science and Technology, Tao-Yuan 33303, Taiwan; 3 Liver Research Center, Department of Hepato-Gastroenterology, Chang Gung Memorial Hospital, Tao-Yuan 33305, Taiwan; chauting@adm.cgmh.org.tw; 4 Department of Anatomic Pathology, Chang Gung Memorial Hospital, Chang Gung University School of Medicine, Tao-Yuan 33305, Taiwan; 5 Graduate Institute of Natural Products, Chang Gung University, Tao-Yuan 33303, Taiwan; 6 Genomic Medicine Core Laboratory, Chang Gung Memorial Hospital, Tao-Yuan 33305, Taiwan; a24255544@gmail.com; 7 Department of Pathology and Laboratory Medicine, Taoyuan Armed Forces General Hospital, Tao-Yuan 32551, Taiwan; 8 Biomedical Engineering Department, Ming Chuan University, Tao-Yuan 33348, Taiwan

**Keywords:** melatonin, hepatocellular carcinoma, miRNA let7i-3p, RAF1

## Abstract

Melatonin is the main pineal hormone that relays light/dark-cycle information to the circadian system. Recent studies have examined the intrinsic antitumor activity of melatonin in various cancers, including hepatocellular carcinoma (HCC), the primary life-threatening malignancy in both sexes in Taiwan. However, the detailed regulatory mechanisms underlying melatonin’s anti-HCC activity remain incompletely understood. Here, we investigated the mechanisms by which the anti-HCC activity of melatonin is regulated. Human hepatoma cell lines were treated with 1 and 2 mM melatonin, and functional assays were used to dissect melatonin’s antitumor effect in HCC; small-RNA sequencing was performed to identify the microRNAs (miRNAs) involved in the anti-HCC activity of melatonin; and quantitative RT-PCR and Western blotting were used to elucidate how miRNAs regulate melatonin-mediated HCC suppression. Melatonin treatment at both doses strongly inhibited the proliferation, migration and invasion capacities of Huh7 and HepG2 cell lines, and melatonin treatment markedly induced the expression of the miRNA let7i-3p in cells. Notably, transfection of cells with a let7i-3p mimic drastically reduced RAF1 expression and activation of mitogen-activated protein kinase signaling downstream from RAF1, and rescue-assay results demonstrated that melatonin inhibited HCC progression by modulating let7i-3p-mediated RAF1 suppression. Our findings support the view that melatonin treatment holds considerable promise as a therapy for HCC.

## 1. Introduction

Hepatocellular carcinoma (HCC) is the sixth most prevalent cancer worldwide and is annually diagnosed in approximately 600,000 people globally [1,2]. In Taiwan, HCC is the leading and the second-leading cause of cancer-related deaths among males and females, respectively. HCC is one of the most challenging cancers to treat, particularly in the late stages of the disease when surgical resection is not possible, and chemotherapy is the only HCC treatment option; however, the patients’ quality of life is adversely affected by the severe side effects of most chemotherapeutic agents. Moreover, even sorafenib, which is accepted as the most efficacious drug targeting HCC, can prolong patient survival by only ~3 months [3,4]. Therefore, the development of early diagnostic markers and new treatment options for HCC represents a critical goal in liver cancer research.

Melatonin is a crucial hormone secreted by the pineal gland that relays light/dark-cycle information to the circadian system [5]. Moreover, melatonin functions in the regulation of various essential events in cellular physiology, including antioxidation, anti-inflammation and immune-cell activation, and is closely related to the occurrence of multiple diseases [6,7,8,9], and melatonin is also produced by a few other tissues besides the pineal gland, such as the liver, skin and small intestine [10,11]. Melatonin regulates cell physiology through receptor-dependent and -independent mechanisms, with the regulation occurring predominantly through the receptor-dependent mechanism: By binding to the receptor MT1 (encoded by *MTNR1A*) or MT2 (encoded by *MTNR1B*), melatonin can activate signaling pathways in cells and regulate physiological processes such as inflammation and the immune response [12,13,14]. Furthermore, melatonin and its metabolites are potent antioxidants that can bind directly to and neutralize peroxides produced in metabolism and thereby reduce cellular damage and act as powerful free-radical scavengers [15,16,17]. Recently, melatonin has also been shown to potentially suppress cancer cell growth and metastasis through distinct mechanisms, and clinical trials have been conducted using melatonin in the treatment of colorectal, lung and breast cancers [18,19,20,21,22]. However, only a few studies to date have addressed the role of melatonin in liver cancer, and the regulatory mechanisms underlying melatonin’s effect in liver cancer remain poorly elucidated.

MicroRNAs (miRNAs) are 21–25 nt-long noncoding RNAs that are widely present in eukaryotes [23,24]. miRNAs regulate gene expression by binding to the 3′ untranslated region (3′UTR) of target mRNAs and thereby inhibiting the translation or promoting the degradation of the mRNAs, which results in the modulation of development, metabolism, cell differentiation, apoptosis and other critical physiological functions [25,26,27,28,29,30]. By affecting mRNA levels in cells, a single miRNA can concurrently regulate several dozen genes, and notably, more than half the genes in the human genome have thus far been shown to be regulated by miRNAs [31]. Furthermore, aberrant miRNA expression has been shown to result in various diseases, including cancer [32,33,34,35,36]; accordingly, several miRNAs are regarded as key targets for disease treatment, and antineoplastic agents such as Taxol and curcumin have been found to regulate miRNAs when used in cancer therapy [37,38].

We have previously shown that melatonin exhibits anti-liver cancer activity, but the mechanistic details of the tumor suppression by melatonin remain incompletely elucidated. Moreover, miRNAs have been shown to play a critical role in liver cancer, but only a few studies have examined the regulatory relationship between melatonin and miRNAs. In this study, we employed small-RNA sequencing to identify the miRNAs that are regulated by melatonin and examined the mechanisms by which melatonin produces its anti-liver cancer effects.

## 2. Results

### 2.1. Melatonin Inhibits HCC-Cell Proliferation, Migration and Invasiveness

**Figure 1:**
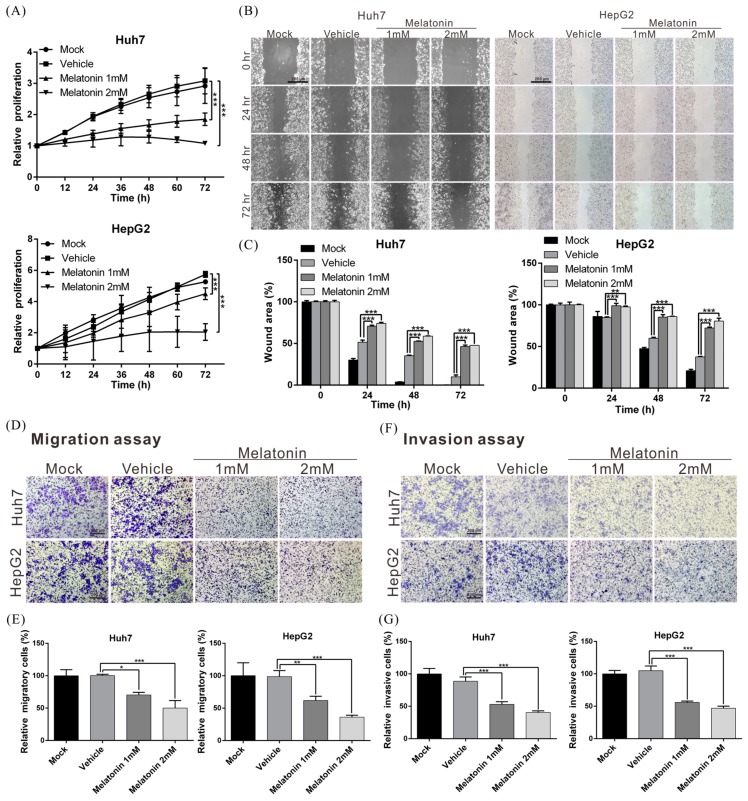
Melatonin suppresses HCC-cell proliferation, migration and invasion capacity in vitro. (**A**) Huh7 and HepG2 cells were treated with/without melatonin. Cell proliferative capacities were examined using an xCELLigence real-time cell analyzer. Mock: cells treated with Dulbecco’s modified Eagle’s medium. Vehicle: cells treated with dimethyl sulfoxide. (**B**) The effect of melatonin on the wound-healing ability of Huh7 and HepG2 cells was examined, and the results were quantified (**C**). Magnification: 100×. Scale bar = 200 μm. (**D**,**F**) Transwell assays were performed to investigate the migration and invasion capacities of Huh7 and HepG2 cells treated with/without melatonin, and the results were quantified (**E**–**G**). All data are expressed as means ± SD of three independent experiments; * *p* < 0.05, ** *p* < 0.01, *** *p* < 0.001. Magnification: 100×. Scale bar = 200 μm.

To investigate the biological role of melatonin in HCC progression, we treated human hepatoma cell line Huh7 and HepG2 cells with 1 and 2 mM melatonin and then measured cell-proliferation rates using an xCELLigence real-time cell analyzer. Melatonin treatment at both doses markedly reduced the proliferation of Huh7 and HepG2 cells as compared with mock and vehicle treatment (Figure 1A). The proliferation of Huh7 and HepG2 cells was suppressed by ~65% and ~64%, respectively, after exposure to 2 mM melatonin.

To measure melatonin’s effect on cell motility, we analyzed cell migration and invasiveness by performing wound-healing and transwell assays. With both cell lines, migration and invasion capacities were substantially lower after melatonin treatment relative to that after vehicle (control) treatment (Figure 1B–G). These results indicate that melatonin exerts tumor-suppressing effects by repressing HCC-cell proliferation, migration and invasion.

### 2.2. Melatonin Induces miRNA Let7i-3p Expression

**Figure 2:**
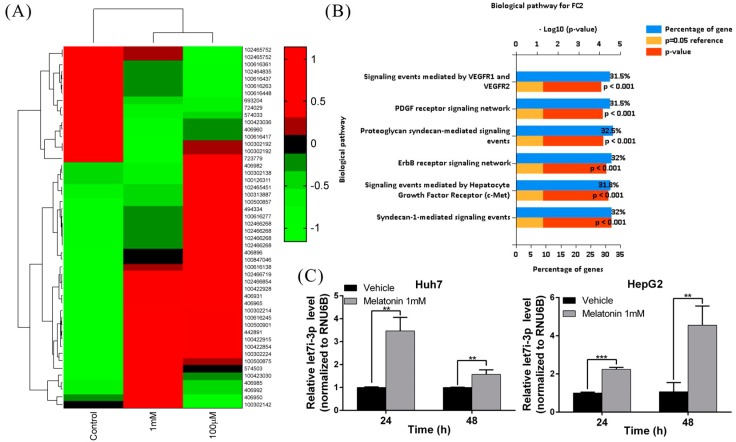
Melatonin induces let7i-3p expression in HCC cells. (**A**) Results of heatmap analysis illustrating miRNA expression profiles in Huh7 cells treated with/without melatonin. (**B**) Bar charts representing the enriched biological pathways associated with the differentially-expressed genes after melatonin treatment. (**C**) Real-time RT-PCR results showing the let7i-3p level after melatonin treatment. RNU6B served as the internal control. Data are expressed as means ± SD of three independent experiments; ** *p* < 0.01, *** *p* < 0.001.

Several drugs have been shown to regulate cellular physiology by modulating the expression of intracellular miRNAs [39,40,41,42]. To determine whether miRNAs are involved in the tumor-suppressing effect of melatonin in the context of HCC, we performed small-RNA sequencing to analyze the miRNA expression profiles of Huh7 and HepG2 cell lines treated with melatonin. The miRNA expression profile of melatonin-treated cells differed significantly from that of control cells (Figure 2A). Next, using Ingenuity Pathway Analysis, we found that melatonin affected the expression of genes associated with angiogenesis and cell-proliferation pathways, such as genes encoding molecules that function in the signaling mediated by the vascular endothelial growth factor (VEGF) receptor, platelet-derived growth factor (PDGF) receptor, ErbB receptor and c-MET (mesenchymal-epithelial transition factor) (Figure 2B). After the results were validated using quantitative real-time PCR, the expression of the miRNA let7i-3p was found to be increased 3–4-fold following melatonin treatment in both cell lines (Figure 2C), which indicated the potential involvement of let7i-3p in the anti-HCC effect of melatonin.

### 2.3. Let7i-3p Acts as a Tumor Suppressor

**Figure 3:**
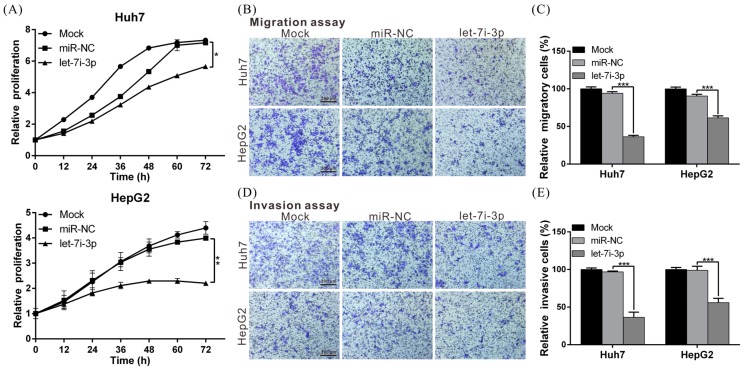
miRNA let7i-3p suppresses the proliferation, migration and invasion capacities of HCC cells. (**A**) Huh7 and HepG2 cells were transfected with 50 nM let7i-3p mimic or control miRNA (miR-NC), and the cell proliferation rate was monitored at the indicated times using an xCELLigence real-time cell analyzer. (**B**,**D**) Transwell assays were performed to examine the effect of let7i-3p on the migration and invasion capacities of HCC cells, and the results were quantified (**C**,**E**). All experiments were performed in triplicate; * *p* < 0.05, ** *p* < 0.01, *** *p* < 0.001. Magnification: 100×. Scale bar = 200 μm.

To elucidate the biological role of let7i-3p, we transfected Huh7 and HepG2 cells with a let7i-3p mimic or control miRNA (miR-NC) and then performed functional assays, which revealed that the proliferation, migration and invasion capacities of let7i-3p-overexpressing cells were significantly lower than those of miR-NC-expressing cells (Figure 3A–E). The results showed that let7i-3p exhibited tumor-suppressor activity in HCC cells and further raised the possibility that melatonin exerts its tumor-suppressing effect by regulating let7i-3p.

### 2.4. Let7i-3p Directly Binds to RAF1 mRNA 3′UTR and Suppresses RAF1 Expression

**Figure 4:**
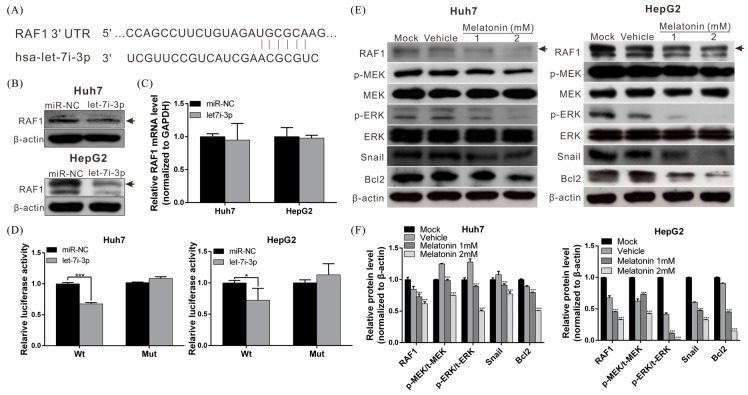
miRNA let7i-3p directly binds to the 3′UTR of RAF1 mRNA and suppresses RAF1 protein expression. (**A**) Schematic representation of the predicted let7i-3p binding region in the 3′UTR of RAF1 mRNA. Western blotting (**B**) and quantitative RT PCR (**C**) were performed to examine the RAF1 level (black arrow) in HCC cells transfected with 50 nM let7i-3p mimic or control miRNA (miR-NC) for 48 h. (**D**) Huh7 and HepG2 cells were cotransfected with pmirGLO-RAF1 reporter plasmids containing the wild-type (Wt) or mutated (Mut) 3′UTR fragments of RAF1 and the let7i-3p mimic or control miRNA (miR-NC), and luciferase activity was analyzed at 48 h after transfection. Data are expressed as means ± SD of three independent experiments; * *p* < 0.05, *** *p* < 0.001. (**E**) Western blotting was used to examine the levels of RAF1 (black arrow) and downstream proteins in HCC cells treated with melatonin for 48 h, and the results were quantified (**F**); *** *p* < 0.001.

We next sought to identify the potential downstream genes regulated by let7i-3p; for this purpose, we used Targetscan miRNA target-detection software, and we identified an evolutionarily-conserved putative binding site for let7i-3p in the 3′UTR of RAF1 mRNA (Figure 4A). Thus, to determine whether let7i-3p represses the expression of endogenous RAF1, we transfected the let7i-3p mimic or scrambled miRNA (miR-NC) into Huh7 and HepG2 cells and then analyzed RAF1 expression by performing Western blotting and quantitative RT-PCR. Our results demonstrated that RAF1 protein level, but not mRNA level, was lower in let7i-3p-transfected cells than in miR-NC-transfected cells (Figure 4B,C).

To ascertain whether let7i-3p directly binds to the 3′UTR of RAF1 mRNA and inhibits RAF1 expression, a RAF1 3′UTR fragment harboring this binding site was cloned downstream of a luciferase-reporter cassette, and the constructed plasmid was transfected into Huh7 and HepG2 cells. An analogous luciferase-reporter construct lacking the putative binding site served as a negative control. In Huh7 and HepG2 cotransfected with the wild-type RAF1 construct and the let7i-3p mimic, luciferase activity was reduced by ~32% and ~28%, respectively, relative to that in the cells cotransfected with the RAF1 construct and miR-NC (Figure 4D). However, the effect of the let7i-3p mimic on luciferase activity was abolished when the putative binding site was deleted in the RAF1 construct. The results indicate that let7i-3p directly bound to the 3′UTR of RAF1 mRNA and suppressed protein translation.

### 2.5. Melatonin Reduces RAF1 Expression and Its Downstream Oncogenic MAPK Signaling

To determine whether melatonin regulates RAF1 expression, we treated Huh7 and HepG2 cells with melatonin for 48 h and then performed Western blotting; our analysis revealed that the RAF1 level was significantly lower in melatonin-treated cells than in vehicle-treated (control) cells. Moreover, the suppression of RAF1 reduced the activation of downstream oncogenic MAPK signaling and the expression of Snail and Bcl2, which resulted in the inhibition of epithelial-mesenchymal transition (EMT) and promoted apoptosis (Figure 4E,F). These results indicate that the mechanism underlying the anti-HCC effect of melatonin involves let7i-3p-mediated RAF1 inhibition and suppression of the oncogenic signaling downstream of RAF1.

### 2.6. Melatonin Inhibits HCC Progression through Let7i-3p-Mediated RAF1 Suppression

**Figure 5:**
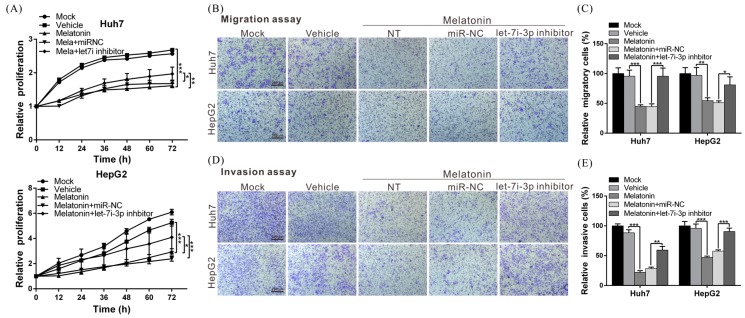
Melatonin exerts its anti-HCC effects through let7i-3p-mediated RAF1 suppression. The inhibitory effects of melatonin on HCC-cell proliferation (**A**), migration (**B**,**C**) and invasion (**D**,**E**) were markedly reversed following treatment with a let7i-3p inhibitor. All experiments were performed in triplicate; * *p* < 0.05, ** *p* < 0.01, *** *p* < 0.001. Magnification: 100×. Scale bar = 200 μm.

To further demonstrate that the anti-HCC effect of melatonin was mediated by the regulation of let7i-3p, we performed rescue assays: we treated Huh7 and HepG2 cells with melatonin and then blocked the function of let7i-3p. Notably, melatonin-induced suppression of cell proliferation, migration and invasion was reversed after blocking let7i-3p (Figure 5A–E). These results confirmed that the anti-HCC effect of melatonin was produced through let7i-3p-mediated RAF1 suppression.

### 2.7. Melatonin Suppresses Tumor Growth In Vivo

**Figure 6:**
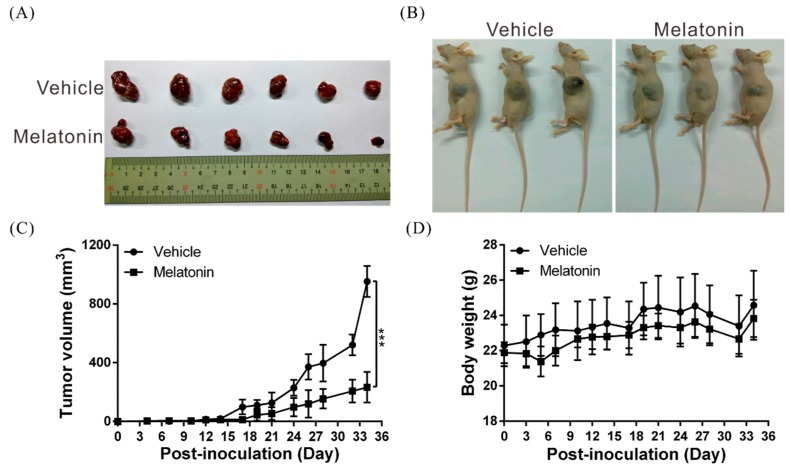
Melatonin suppresses tumor growth in vivo. (**A**,**B**) A total of 5 × 10^6^ Huh7 cells were subcutaneously implanted into the left and right flank regions of mice, and the mice were divided into two groups (*N* = 6/group); one group was injected with 100 µL of melatonin (at a dose of 40 mg/kg body weight), and the other was injected with an equal volume of vehicle (dimethyl sulfoxide), five days per week. Here, representative images of the tumor xenografts at four weeks after implantation are shown. (**C**) Tumor volumes were calculated every three days after injection. The volume of each tumor was calculated thusly: volume = length × width^2^ × 0.5. Bars indicate SD; *** *p* < 0.001. (**D**) Body weights were determined every three days after injection.

Lastly, we conducted studies to validate the tumor-suppressing effects of melatonin in vivo. First, we established a xenograft mouse model and examined tumor growth in these mice. In agreement with the results obtained with the cell model, tumor growth rate was markedly lower in melatonin-treated mice than in control mice (Figure 6A–C). Furthermore, the melatonin-treated and control mice showed no statistically-significant differences in body weight, which suggests that melatonin did not exert toxic effects (Figure 6D).

**Figure 7:**
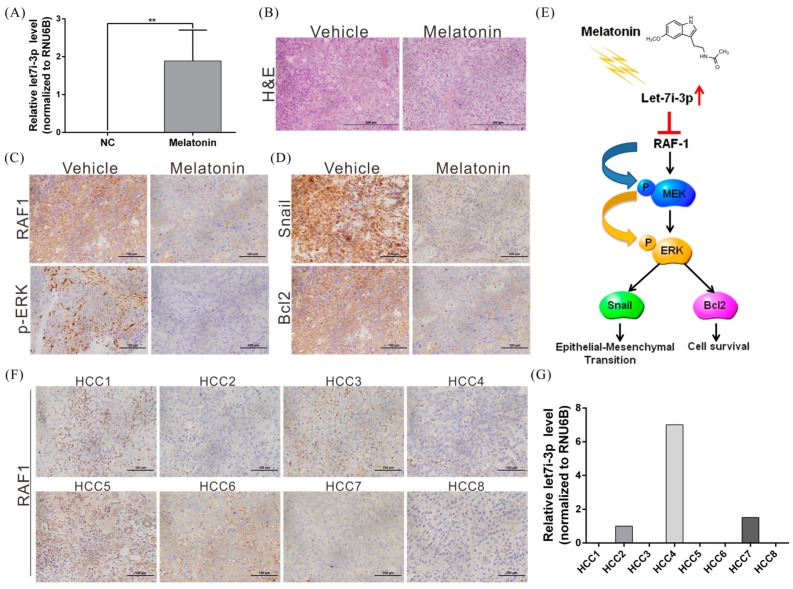
Melatonin induces let7i-3p expression and thereby suppresses RAF1 and its downstream oncogenic pathway. (**A**) Xenograft tumors were excised from mice at the end of experiments and subject to real-time PCR for detecting let7i-3p expression; ** *p* < 0.01. (**B**) Hematoxylin and eosin (H&E) staining was performed to analyze tissue morphology. (**C**,**D**) Immunohistochemical staining showing the effect of melatonin on RAF1, phospho-ERK (P-ERK), Snail and Bcl2 protein expression in mouse xenograft tumors. Magnification: 400×. (**E**) A schematic summarizing the mechanism by which melatonin suppresses HCC progression. (**F**,**G**) Inverse relationship between let7i-3p and RAF1 expression in tissues from patients with HCC. Scale bar = 100 μm.

Second, we verified the regulatory correlation between melatonin, let7i-3p and RAF1 by performing immunohistochemical analyses. Whereas let7i-3p expression was significantly induced in the tumors of mice treated with melatonin, the levels of RAF1, phospho-ERK, Snail and Bcl2 were decreased, which indicates that melatonin affects let7i-3p and the downstream RAF1 signaling pathway (Figure 7A–E).

Third, we analyzed the expression of let7i-3p and RAF1 in tissues of eight patients with HCC; our results showed that let7i-3p and RAF1 levels showed a negative correlation trend (Figure 7F,G). These findings further confirm the tumor-suppressor role of let7i-3p and its regulatory relationship with RAF1.

## 3. Discussion

The RAF1-MEK-ERK signaling pathway performs critical functions in regulating normal development, the cell cycle, cell growth, differentiation and survival [43,44]; thus, aberrant activation of this signaling pathway or abnormal expression of the molecules in the pathway leads to uncontrolled cell growth and therefore multiple diseases, including cancers. Recently, RAF1 overactivation has been shown to occur in various cancers (e.g., breast, lung and thyroid cancers) and is regarded as a critical marker in cancer treatment [45,46,47]. Accordingly, several RAF-targeting small-molecule inhibitors, such as sorafenib, have been employed in cancer treatment, and these have demonstrated high efficacy [48]. Here, we have shown for the first time that melatonin induces the expression of the miRNA let7i-3p in HCC cell lines and have further demonstrated that let7i-3p directly inhibits RAF1 protein translation and activation of the RAF1 downstream oncogenic pathway, thereby suppressing the ability of HCC cells to grow and metastasize and promoting their apoptosis (Figure 7E). Our results indicate that melatonin holds considerable potential for use in the treatment of cancers exhibiting RAF1 overactivation.

miRNAs are widely recognized to play critical roles in diverse cellular processes, and more than half of all human genes have been found to be regulated by miRNAs. Furthermore, numerous therapeutic agents exert anticancer effects by modulating the expression of intracellular miRNAs [38,49,50]. Here, through small-RNA sequencing analysis, we identified 57 miRNAs showing >2-fold differential expression following melatonin stimulation, which indicated that some of these miRNAs contribute to the anti-HCC effect of melatonin. Intriguingly, the results of rescue experiments demonstrated that when we used an inhibitor to block the function of let7i-3p, the anti-HCC effect produced by melatonin was reversed markedly, but not completely. This finding suggests that other molecules or miRNAs are also involved in melatonin’s regulation of cancer, although further investigation is necessary to confirm this.

Previous studies have indicated that melatonin regulates cell physiology primarily through the receptor-dependent pathway [51]. Accordingly, in studies on breast and colorectal cancers, cancer cell lines expressing MT1/MT2 receptors at low levels were found to show low sensitivity to melatonin treatment [52,53]. Here, we found that treatment with the same concentration of melatonin suppressed the growth and migration of Huh7 and HepG2 cell lines to distinct levels, which, we speculate, could be due to different levels of MT1/MT2 receptor expression in these liver cancer cells. However, further studies are required to identify the specific receptors through which melatonin transmits its anti-liver cancer signals and to uncover the detailed underlying signaling pathways and regulatory mechanisms. This also represents the direction for the next stage of our research on HCC.

Melatonin, a hormone secreted by the pineal gland, has been increasingly reported to exhibit anticancer activity; however, few studies to date have addressed the regulatory correlation between melatonin and miRNAs. Our results indicate that by inducing the expression of let7i-3p, melatonin can potentially downregulate the expression of RAF1 and the activation of the oncogenic pathway downstream from RAF1, and thereby suppress the growth and metastasis of liver cancer cells. Furthermore, our animal studies revealed that melatonin is not cytotoxic, and melatonin could, therefore, help improve patients’ quality of life if used in the treatment of liver cancer. Our clarification here of the regulatory network involved in melatonin’s suppression of cancer should facilitate the selection of the most suitable patient groups for drug treatment and serve as a reference for melatonin use in combination with other anticancer drugs.

## 4. Material and Methods

### 4.1. Human Specimens 

Human formalin-fixed paraffin-embedded (FFPE) tissues used in this study were obtained from 8 HCC patients who underwent surgical resection at Chang Gung Memorial Hospital (Tao-yuan, Taiwan) between 2010 and 2015. To reduce the differences between individuals, patients of the same sex and similar age and acquisition time were selected to rule out the influence of these factors. This study was approved by the Ethics Committee of Chang Gung Memorial Hospital (IRB Approval No. 201700439B0; approval date: 30 March 2017), and written informed consent was obtained from each patient.

### 4.2. Cell Lines, Antibodies, miRNAs and Plasmids

The HCC cell lines HepG2 and Huh7 were purchased from American Type Culture Collection (Manassas, VA, USA) and maintained in Dulbecco’s modified Eagle’s medium (DMEM) containing 10% fetal bovine serum at 37 °C in a 5% CO_2_ atmosphere. Polyclonal antibodies against RAF1, MEK, ERK, Bcl2 and β-actin were purchased from GeneTex (Irvine, CA, USA) and Abcam (Cambridge, UK), respectively. Secondary antibodies were purchased from Santa Cruz Biotechnology Inc. (Santa Cruz, CA, USA). The commercialized let7i-3p mimic and inhibitor were purchased from Thermo Fisher Scientific (Waltham, MA, USA). The plasmid pmirGLO-RAF1-WT, a PGK-based expression plasmid containing full-length RAF1 3′UTR (nt 211–543), and an analogous luciferase-reporter construct lacking the putative let7i-3p binding site (pmirGLO-RAF1-mut) were constructed by GenScript Co. (Piscataway, NJ, USA).

### 4.3. Cell Proliferation Assay

The effect of melatonin on cell proliferation was monitored using an xCELLigence real-time cell analyzer (Roche Life Science, Indianapolis, IN, USA), as previously described [54]. Briefly, 3 × 10^3^ Huh7 or HepG2 cells were subcultured in 96-well E-plates and cultured in DMEM in the presence or absence of melatonin. The impedance value of each well was automatically monitored by the xCELLigence system for a duration of 72 h and expressed as a CI (cell index) value.

### 4.4. Cell Migration and Invasion Assays

We examined how melatonin affects HCC cell migration and invasion by performing wound-healing and transwell assays, as previously described [55].

### 4.5. RNA Isolation

Total RNA was extracted using TRIzol^®^ Reagent (Invitrogen, Carlsbad, CA, USA), according to the manufacturer’s instructions. The purified RNA was quantified by measuring the 260-nm absorbance using an ND-1000 spectrophotometer (Nanodrop Technology, Waltham, MA, USA), and RNA quality was assessed using a Bioanalyzer 2100 (Agilent Technology, Santa Clara, CA, USA) with an RNA 6000 LabChip kit (Agilent Technologies).

### 4.6. Library Preparation and Sequencing

Small-RNA library construction and deep sequencing were performed at a biotechnology company (Welgene, Taipei, Taiwan). Samples were prepared using an Illumina sample preparation kit, according to the TruSeq Small RNA Sample Preparation Guide. The 3′ and 5′ adapters were ligated to total RNA, and the RNA was reverse-transcribed and PCR-amplified. The enriched cDNA constructs were size-fractionated on 6% PAGE gels, and the bands corresponding to 18–40-nt RNA fragments (140–155 nt in length with both adapters) were excised for purification. Libraries were sequenced on an Illumina instrument (75SE cycle single read), and sequencing data were processed using Illumina Pipeline software bcl2fastq v2.0 (Illumina, San Diego, CA, USA).

### 4.7. Small-RNA Sequencing Analysis

Initially, the generated sequences were filtered to obtain qualified reads. Trimmomatic was implemented to trim or remove reads according to the quality score. Qualified reads remaining after filtering out low-quality data were analyzed using miRDeep2 to clip the 3′ adapter sequence and discard reads that were <18 nt long, and then, the reads were aligned to the human genome sequence from UCSC (University of California, Santa Cruz) Genome Browser. Only reads that mapped correctly to the genome ≤5 times were used for miRNA detection because miRNAs typically map to few genomic locations. miRDeep2 estimates the expression levels of recognized miRNAs and also identifies previously unknown miRNAs.

### 4.8. Detection of MiRNA Let7i-3p through Quantitative Real-Time RT-PCR

Huh7 and HepG2 cells were treated with/without melatonin for 48 h, and then, cellular miRNAs were isolated using a miRNeasy Mini Kit (QIAGEN, Gaithersburg, MD, USA), according to the manufacturer’s instructions. The miRNAs were converted into cDNA with a TaqMan microRNA Reverse Transcription Kit (Thermo Fisher Scientific) and then subject to quantitative real-time RT-PCR for detection of miRNA let7i-3p expression using the TaqMan microRNA assay (Thermo Fisher Scientific); RNU6B was used as an internal control.

### 4.9. Estimation of Protein Levels through Western Blotting

Huh7 and HepG2 cells were treated with 1 and 2 mM melatonin for 24 and 48 h, washed twice with phosphate-buffered saline (PBS) and then lysed in 200 μL of RIPA lysis buffer (BIOTOOLS Co., Ltd., New Taipei City, Taiwan) containing protease inhibitors. Next, total protein (100 μg/lane) from the supernatant was loaded onto SDS-polyacrylamide gels for Western blotting analysis to detect the levels of RAF1, MEK, ERK, Bcl2 and EMT-related proteins. Immunoreactive bands were visualized using an enhanced chemiluminescence system (NEN Life Science Products, Boston, MA, USA) and developed using Kodak X-ray films. The volume of each band was quantified using ImageQuant 5.2 (GE Healthcare, Piscataway, NJ, USA).

### 4.10. Transient Transfection of the pmirGLO-RAF1 Plasmid and miRNAs

HepG2 and Huh7 cells were seeded in 6-well plates at a density of 3 × 10^5^ cells/well, cultured for 24 h and then transfected with 0.5 μg of the constructed pmirGLO-RAF1 plasmid by Lipofectamine 3000 (Invitrogen) according to the manufacturer’s instructions. At 6 h after transfection, the cells were transfected with 50 nM synthetic let7i-3p mimic or inhibitor, and after another 48 h, the cells were harvested and used in luciferase assays.

### 4.11. Mice

Male 6–8-week-old nude mice (BALB/cAnN-Foxnlnu/CrlNarl) were purchased by the National Laboratory animal center (Taipei, Taiwan) and housed in a pathogen-free environment under a 12/12-h light/dark cycle and fed autoclaved standard chow and water. Mice were bred at the Animal Center of Chang Gung Hospital (Tao-Yuan, Taiwan) in accordance with the Guidelines for the Care and Use of Laboratory Animals (National Institutes of Health, NIH). All experiments related to the animal studies were approved by the Institutional Animal Care and Use Committee (IACUC) at Chang Gung Hospital (IACUC Approval No. 2017032205; approval date: 24 May 2017).

### 4.12. In Vivo Tumor Growth Assay

Huh7 cells (5 × 10^6^) were resuspended in 100 μL of saline and injected subcutaneously into the left and right flank regions of mice. All tumors were staged for 1 week before initiating drug treatment. Starting from the beginning of the second week, the mice harboring tumors were intraperitoneally injected with 100 µL of melatonin (at a dose of 40 mg/kg body weight) or an equal volume of vehicle (dimethyl sulfoxide; used as a control) for 5 days per week. Melatonin was administered 1 h before the room lights were switched off. Tumor volumes were calculated using the formula V = length × width^2^ × 0.5.

### 4.13. Immunohistochemistry

Slides bearing tissue sections for immunohistochemistry were deparaffinized and then rehydrated through a graded ethanol series. For antigen retrieval, slides were boiled in Trilogy reagent (Cell Marque, Rocklin, CA, USA) for 10 min, allowed to cool at room temperature for 30 min, washed with 1× PBS and then immersed in 3% hydrogen peroxide for 10 min to block endogenous peroxidase activity. After three rinses with 1× PBS, sections were exposed to primary antibodies (Santa Cruz Biotechnology Inc.) for 1 h at room temperature, washed thrice with 1× PBS and then incubated with biotinylated secondary antibodies (Dako, Glostrup, Denmark) for 25 min. After another three rinses with 1× PBS, sections were incubated with horseradish peroxidase-conjugated streptavidin for 25 min at room temperature, and then, the peroxidase activity was detected using a DAB substrate chromogen (Dako) at room temperature. Lastly, sections were counterstained with hematoxylin and eosin.

### 4.14. Data Analysis

All assays were repeated thrice. All statistical analyses were performed using SPSS 16.0 software (SPSS Inc., Chicago, IL, USA). The statistical tests were all two-sided, and *p* < 0.05 was considered significant. Data are expressed as means ± SD. Experiments were performed at least 3 times.
